# Patient, Parent, and Provider Perceptions of Barriers to Pediatric Inflammatory Bowel Disease Care

**DOI:** 10.1097/PG9.0000000000000386

**Published:** 2023-11-08

**Authors:** Jennifer L. Dotson, Josh Bricker, Deena J. Chisolm, Laura M. Mackner

**Affiliations:** From the * Division of Pediatric Gastroenterology, Hepatology and Nutrition, Nationwide Children’s Hospital, Columbus, OH; † The Center for Child Health Equity and Outcomes Research, The Research Institute, Nationwide Children’s Hospital, Columbus, OH; ‡ The Center for Biobehavioral Health, The Research Institute, Nationwide Children’s Hospital, Columbus, OH.

**Keywords:** barriers to care, children, focus group

## Abstract

**Objectives:**

Children with inflammatory bowel disease (IBD) have a significant life-long burden as a result of disease, impacted by environmental and individual barriers. Successful health system interventions require a comprehensive approach, informed by various stakeholders. The main objective was to identify health system barriers and potential solutions from existing patients, families, and providers via focus groups.

**Methods:**

Participants for the focus groups were existing English-speaking patients (ages 9–18) with IBD, their caregiver(s), and providers including multiple professions (eg, physician, nurse, pediatrician, social worker, care coordinator, scheduler, and psychologist). Separate focus groups were led by experienced personnel for parents, children, and providers, using a standardized interview guide. Sessions were recorded, transcribed, and verified. Using content analysis, we systematically classified data through coding and identified themes.

**Results:**

Focus groups comprised (a) 3 patient groups (n = 20, 50% female, including 2 younger; mean age = 11.4 ± 1.5 years) and 1 older group (mean age = 15.6 ± 1.3 years), (b) 3 parent groups (n = 24, 83% female), and (c) 2 multidisciplinary provider groups (n = 19). Families shared several common concerns with providers (eg, school, care delay, psychosocial, and financial) but varied on specifics. Some barriers may be addressable through family or staff education, improved communication (eg, care delay/ access, transition), or training (eg, labs and diet), while others may require change at an institutional or policy level (eg, insurance).

**Conclusions:**

This qualitative analysis identified several barriers to IBD care, some shared, some unique to patients, parents, and providers, highlighting the importance of obtaining multiple stakeholder perspectives when exploring barriers to care.

**What is known**

- Significant healthcare barriers exist for children with chronic diseases.
- Healthcare barriers contribute to ineffective care and poor health outcomes.

**What is new**

- This qualitative analysis identified several barriers to inflammatory bowel disease (IBD) care among multistakeholder groups, including the importance of psychosocial issues when caring for children and adolescents with IBD.
- Psychosocial and school-related issues were reported as barriers to healthcare. Factors related to the experience of the disease such as these have not previously been reported as healthcare barriers.

## INTRODUCTION

Significant healthcare barriers exist for children with chronic diseases such as inflammatory bowel disease (IBD) and contribute to ineffective care and poor health outcomes. Commonly identified barriers include low socioeconomic status, limited access to care, health care system inefficiencies, low health literacy, and poor adherence to medical advice or medication (1–3). Health system barriers and hardships may contribute to lower quality of life (QOL) and suboptimal disease outcomes (4–6). Data suggest that adolescents with IBD who have increased disease severity and lower adherence are associated with higher barriers as well as and higher anxiety/depressive symptoms (7,8).

In other chronic conditions, healthcare barriers affecting children have been associated with increased hospital readmissions (9), symptoms, perceptions of greater illness severity (10,11), and worse clinical outcomes (12). The current literature lacks data regarding the physician- and patient-perceived barriers in pediatric IBD.

The study goal was to identify health system barriers and potential solutions from existing patients, families, and providers via focus groups. Physician- and patient-perceived barriers may differ from one another, so any successful health system intervention requires a comprehensive approach informed by multiple perspectives throughout the course of a child’s illness. We conducted separate focus groups asking each group to identify and discuss *all* barriers impacting IBD care.

## METHODS

Youth with IBD were recruited prospectively. English fluency was required to effectively participate in discussions. Planned focus groups included 2 younger child groups (9–13 years), an older child group (14–18 years), 3 groups of corresponding parents, and 2 multidisciplinary provider groups.

Given focus groups were conducted in the evening. Patients were selected from a local IBD database primarily living within an hour drive of the hospital. We contacted 156 families via telephone and clinic visits. Seventy-four (47%) families responded. Of these, 21 (28%) agreed to participate, 6 tentatively agreed, and 47 declined. Reasons for decline included being too busy (32%), specific conflicts with focus group dates (6%), lack of interest/no specific reason given (62%). Provider groups (Nationwide Children’s Hospital-affiliated [NCH]) were recruited via email and included representatives from key specialties involved with IBD care, including a community pediatrician, pediatric gastroenterologists, surgeon, nurses, administrative assistant, social worker, dietitian, care coordinator, nurse practitioner, scheduler, and psychologist.

Sessions occurred in conference rooms and were allotted 1–2 hours for discussions. The faculty facilitators were a coauthor (DC) and the director of the Biobehavioral Outcomes Core at NCH. Both have expertise with qualitative methodology and conducting focus groups. Neither are involved with direct patient care of study participants, which allows participants to freely offer their own ideas and share experiences. After introductions and ice-breaker exercises, the discussion facilitator prompted discussion with standard entreaty to share barriers faced in obtaining health care. A standardized discussion guide was developed by the study team and used for each focus group (see Supplemental Digital Content 1, http://links.lww.com/PG9/A149). The questions for the discussion guide were developed based on focus group methodology, the expertise of the study team, and previous research on barriers in healthcare. The lead author developed the first draft of the guide which was reviewed and revised by the other authors. The core of each discussion guide was the same with slight modifications to the verbiage and ice-breaker questions to tailor to each audience. Participants were asked about barriers to their health care, medical care, and IBD care in different prompts. Sessions were audio recorded, transcribed verbatim, and verified by study staff.

The study was approved by the NCH IRB and conducted prior to the COVID-19 pandemic. Patient and caregiver participants were given $25.00, meal, and parking voucher. Medical team participants were provided a meal. Informed consent/assent was obtained from all participants.

Transcripts were analyzed by two authors (JD, JB) using iterative content analysis through a process of coding and generating themes through a systematic classification process with an a priori focus on health care barriers and solutions until saturation of themes was achieved. They systematically classified parts of each discussion (by sentence, group of sentences, or paragraphs) to represent a theme related to health care barriers or solutions. A running list of themes and subthemes was updated and revised as necessary. Coding schemes developed by each coder were discussed and consensus was reached on a final set of codes to represent all focus group data. A third author (LM) was available for consultation when needed.

## RESULTS

Experienced research personnel conducted eight focus groups including three parent groups (n = 24, 83% female), three child patient groups (n = 20, 50% female), and two multidisciplinary provider groups (N = 19) over the course of two months. The child groups included two younger child groups (M age = 11.4 ± 1.5 years) and one older child group (M age = 15.6 ± 1.3 years). Recorded session transcripts lasted 49–84 minutes (group means: 60 minutes for children, 73 minutes for parents, 75 minutes for providers). A complete list of barrier themes and potential solutions are summarized in Tables 1 and 2. Several barrier themes which included select representative quotes are subsequently discussed.

**TABLE 1.** Main barrier themes according to patients, parents, and providers

Themes	Patients	Parents	Providers
Care delay/access	X	X	X
Care variation	X	–	X
Communication	X	X	X
Coping	X	–	–
Diagnostics/testing	X	–	–
Diet	X	X	–
Education/information sources	X	X	X
Infusion	X	X	X
Labs	X	X	–
Psychosocial	X	X	X
School/504	X	X	X
Medications	X	X	–
Transition/continuity of care	X	X	–
Financial	–	X	X
Insurance	–	X	X
Scheduling	–	X	X
Treatment	–	X	–
Resources/staffing	–	–	X
Transportation	–	–	X

**TABLE 2.** Summary of barrier themes and potential solutions for each stakeholder group

Themes		Patients	Parents	Providers
**Care delay/access**	Barrier	• Misdiagnosed, or urgency of concerns/symptoms dismissed	• Misdiagnosed, or urgency of concerns/symptoms dismissed • Delayed hospital admission	• Family treatment decision delay • Family delay in reporting symptoms • Medication delay due to insurance and pharmacy
	Solution	• None identified	• Primary care provider should listen to their concerns • Increase local pediatric IBD care	• None identified
**Care variation**	Barrier	• Inconsistent care for chronic illness in ED	• None identified	• Variation in decision to send patient to ED • Variation in ED and pediatric GI expertise
	Solution	• None identified	• None identified	• None identified
**Communication**	Barrier	• Privacy not taken seriously • Irrelevant questions • Not speaking to kids at their level of understanding	• MyChart restrictions and limitations • Nurse insensitive to patient • Doctor not respecting parent’s opinion • Delayed or inefficient communication	• Challenges in motivating family to accept medications as treatment • Awkwardness when asking about adherence • MyChart limitations
	Solution	• Medical team should speak in “kid’s language”	• MyChart usage and timely responses	• Increase MyChart usage • Foster relationships between primary care and various subspecialists (eg, education and social events)
**Diagnostics/ Testing**	Barrier	• Difficulty ingesting contrast (MRI) and bowel prep (colonoscopy) • Discomfort with idea of colonoscopy	• None identified	• None identified
	Solution	• More information about procedures	• None identified	• None identified
**Diet**	• Barrier	• Dietary restrictions	• Doctors’ “pushing medication” approach versus parents’ trust in nutritional solutions • Bad experience with enteral therapy	• None identified
	• Solution	• More healthy food options at school	• Clear nutrition recommendations/ resources	• None identified
**Education/information sources**	• Barrier	• Doctors not educated about alternative medicine and healing foods • Doctors did not discuss treatments enough • Unhelpful information from dietician	• Want more information related to disease and treatment options • Lack of centralized source for information about resources (financial, family support, etc.)	• Families may not be receptive to learning at diagnosis • How to educate families about the disease • How to motivate to take medication • Competing with less reliable information on the Internet
	• Solution	• None identified	• Standardized information/checklist given to all families at diagnosis (resource hub – webpage, MyChart) • Newsletter	• Ongoing disease education
**Infusion**	• Barrier	• Long wait times • Pain/discomfort from inserting and removing IV • Lethargy from diphenhydramine	• Wait and travel time • Concern for medication immediate and long-term effects	• Difficulty coordinating rapid infusion and GI clinic appointments • Concern for family travel and infusion time • Limitations on billing for physician time, inpatient bed (for rapid infusion)
	• Solution	• Rapid infusion protocols	• Infusions options closer to home • Rapid infusion protocols	• Increase infusion clinic flexibility/options • Rapid infusion protocols
**Labs**	• Barrier	• Blood draw mishaps, unkind, or inexperienced nurses • Wait time; finding time to go	• Delays in lab results, especially before clinic appointments	• None identified
	• Solution	• Numbing medications	• MyChart ability for outside lab results and notifications when lab orders are placed	• None Identified
**Psychosocial/ Coping**	• Barrier	• Feeling awkward or embarrassed about disease, taking meds, frequent bathroom use • Feeling that kids at school are intrusive with questions; bullying, or spreading rumors • Symptoms affecting sports participation, school, sleep • Coping with symptoms • Joint and stomach pain and sleeplessness	• Caregiver stress; lack of social support for parents • Bullying; lack of social support for child	• Limited community, hospital resources • Family living and social situation • Families who are irresponsible, do not self-advocate • Patients who are in denial, unmotivated, fear negative attention
	• Solution	• Seeking help from guidance counselors	• Mentor/support/ways to meet others	• Routinely ask about the parents’ psychosocial functioning
**School/ 504**	• Barrier	• Difficulty getting permission from teachers to use bathroom • Intrusive questions from teachers about disease	• Child using 504 status as an excuse • Disease affecting college choice and ability to attend	• Need to educate some school leaders and some teachers about disease • Parents who do not authorize communication with school • Kids who misuse 504 status
	• Solution	• More education/ awareness/ understanding by school nurse and teachers	• Awareness/help with accommodations for college (disabilities office)	• None identified
**Medications/ Treatment**	• Barrier	• Aversion to taking medications • Body conscious due to side effects • Social stigma from taking medications • Interaction with OTC medications • Planning where to take medications • Parents’ nagging reminders	• Mail order prescription delays • Pharmacy refills require that patient is on last pill • Feeling pressure from doctor on course of treatment they have reservations about • Not understanding physician recommendations	• None identified
	• Solution	• Pharmacies would fill all prescriptions on the same day • Tips/strategies to help with taking pills • Phone reminders/ pill boxes/ plan for the day around activities	• None identified	• Ongoing disease/medication education
**Transition/continuity of care**	• Barrier	• Starting over with medications in transition to adult GI doctor • Feeling immature to take over responsibility for own care	• Concern about child’s ability to take more responsibility for own care • Transition to different child GI physician • Transition to adult GI physician	• None identified
	• Solution	• None identified	• Transition process/checklist	• None identified
**Insurance**	Barrier	• None identified	• Denial of effective treatments and medications, gaining approval requires multiple calls, steps, delays	• Slow, complex claim approval process • Insurance company dictating care • Disagreement among providers on value of insurance “case manager”
	Solution	• None identified	• Fix the insurance/systemic issues	• Case manager/advocate to help navigate • Encourage families to advocate with insurance companies • Fix the insurance/systemic issues (eg, advocacy, vote, write letters)
**Scheduling**	Barrier	• None identified	• Limited convenient appointment times • Restrictions on scheduling next appointments	• Families who do not return calls • Families need to be more flexible to available times or change providers • Disagreement on how flexible physicians should be with appointment times • Scheduling process inefficient
	Solution	• None identified	• Scheduling appointments further in advance	• Increase flexibility with scheduling appointments (eg, scheduling, days/times, telehealth) • Schedule at time of visit during check out
**Financial**	Barrier	• None identified	• Significant out-of-pocket costs • Concern about future cost of care	• Treatment costs • Middle class families not qualifying for incentive programs for established medications • Parents cannot afford time off work for multiple visits • Providers reluctant to ask about finances (eg, making parent uncomfortable, patient may fear not getting best care) • Patients differ in disclosing finances (some admit unaffordable, some self-conscious)
	Solution	• None identified	• Awareness about secondary insurance provider options • Case manager/advocate	• Case manager/advocate to help navigate
**Resources/ staffing**	Barrier	• None identified	• None identified	• Insufficient staff • Limited time to educate each patient about insurance benefits, limitations, pitfalls
	Solution	• None identified	• None identified	• More resources/staff (eg, social worker and psychology support)
**Transportation**	Barrier	• None identified	• None identified	• Some patients lack transportation for visits • Too time-consuming for patients with Medicaid to use transportation benefit
	Solution	• None identified	• None identified	• Parking/gas vouchers

The phrase “None identified” indicates a group did not discuss a specific barrier related to that theme or did not offer a solution to the identified barrier within the corresponding theme.

GI = gastroenterology; MyChart = electronic patient portal; MRI = magnetic resonance imaging.

### Care Variation

Providers discussed variation in sending patients to the emergency department (ED), variability in local ED resources and expertise, and treatment variation due to suboptimal standardization.

…there’s not that much you can do in the office. When I talk to them on the phone that is not always clear…I would have been overwhelmed dealing with that issue (in the office).

### Communication

Patients expressed a desire for physicians to communicate clearly and expressed confidentiality concerns. Parents were frustrated with MyChart’s restrictions (eg, patient’s approval for access, limitations, response timeliness). Some felt insensitivity or disrespect in communications with medical teams.

...they would cut me off with what I’d try to tell them, and I got to the point where I’m just going to keep my mouth shut because you know everything…

### Education/Information Sources

Parents wanted to be educated about the disease and treatment options at diagnosis, and they sought information online. Parents expressed interest in receiving comprehensive information (eg, resource hub and newsletters) and regret not knowing information sooner. Providers struggled with how to educate families when “sometimes they are just not mentally or emotionally ready to hear a lot of the information” and the need for ongoing education/re-education.

Some of my more anxious families…are looking at out-of-the-box, homeopathic, dietary options...gets in the way of getting good care and following the doctor’s recommendations

### Psychosocial/Coping

Patients reported that peers at school asked intrusive questions, sometimes bullying or spreading rumors.

…I feel kinda, like, bullied about it...kids are, like, talking behind my back, and they’re always asking me why I take so long (in the bathroom)

Some felt guilty for expressing anger while in pain or when a parent “missed out” on something because they were not feeling well. Parents described employment burdens and difficulty balancing professional and caregiver roles. Patients suggested seeking help from school counselors, and parents preferred additional support opportunities (eg, mentoring, support groups, education activities). Providers identified multiple psychosocial barriers to care including limited community resources, limited parental psychosocial screening, social or living situation, and lack of self-advocacy with insurance companies. They suggested increasing psychosocial screening of parents/caregivers to help identify needs.

### School/504

Patients reported difficulty asking to use the restroom during class with varying degrees of resistance by teachers, defending the duration of time in the bathroom or were met with intrusive questions afterward.

…some of my teachers are strict...I told her I needed to go real bad, and she already knew I had IBD, and then I didn’t go till lunch time, so, and then, you know...I had an accident.

Patients felt that increased IBD education could improve awareness and understanding among teachers and school staff. Providers noted issues when school leaders/teachers are unfamiliar with the disease and unsympathetic to the child’s needs.

…getting punished in classroom for leaving without a bathroom pass... it’s put on the child’s back to advocate for themselves, which can be very challenging…

### Transition/Continuity of Care

One patient expressed concerns about where to receive care when in college. Another recounted his brother’s experience in which an adult GI physician “wanted to take him off all his medications, and he didn’t like that decision because he’d spent like 6 years trying to get balanced out so he could go to college.” Parents were uneasy over transitioning care and disrupting the treatment regimen. Parents were concerned about their child’s ability to manage their disease with some emphasizing the importance of gradually increasing responsibility and suggested a transition process and using a skills checklist.

There is no way (she is) going to get on the phone and make sure her Humira gets here...

### Insurance

Several parents shared stories of insurance denials for procedures or medications. Parents who were able to tackle insurance barriers became more aware of the various rules of coverage and reasons for denial or were willing to seek assistance from others to navigate the process.

It’s always rejected until you fight for something, and if you don’t have time, or if you don’t have the intelligence, and you don’t have the patience to do all that, I don’t know what happens.

Providers identified several insurance barriers, including the tendency to “dictate how (a given) therapy is delivered” and which labs or treatments are “medically necessary”, which requires extra work to justify treatment decisions.

…prior authorizations and approvals and all of the paperwork that goes along with it is really a huge…incredible barrier to care.

Parent and providers agreed there is a critical need to systematically fix insurance and healthcare delivery. Concrete solutions were not identified for this complex problem, but they emphasized importance of advocacy and voting, and perhaps a case manager/advocate to help navigate families through it.

### Scheduling

Parents found scheduling limitations frustrating and inconvenient (“…he was missing the same classes in school every time”).

Providers identified several scheduling barriers including family communication and flexibility, physician flexibility, and inefficiencies in the scheduling process. Physicians differed in their willingness to accommodate family schedules-whether out of convenience or more urgently to keep them out of the ED. Both groups agreed that increased flexibility within the scheduling process and types of visits could help (eg, scheduling, days/times, and telehealth).

## DISCUSSION

This qualitative analysis identified multiple health system barriers to IBD care common to families and providers. Several barriers have previously been identified in the literature, such as insurance and financial concerns, transportation, staffing, and education. However, some barriers to health care that were identified are related to the experience of the disease, such as psychosocial and school-related issues. IBD affects multiple aspects of functioning, and many of these areas are viewed as barriers to health care by patients and families. As such, this remains a pervasive and persisting issue for our patients and is the focus of this discussion.

While psychosocial issues may not be a tangible barrier such as financial concerns, they significantly impact health care and outcomes (13). In this study, social concerns for patients such as difficulty managing social situations, bullying, and difficulty participating in sports were identified as barriers, as well as poor social support for both caregivers and patients. Patients and parents described daily burdens of living with IBD and caregiver stresses. Providers drew upon their various experiences and reflected on perceived barriers for their patients and families including community services, difficult living situations, denial, fear, and limited advocacy experience. A practical solution for patients and families could involve a robust partnership with the school and guidance counselors to assist with navigating the school and peer environment.

Parents suggested that mentoring or other ways to meet supportive peers could be a solution, and in fact a peer mentoring program for youth with IBD has been developed and is currently being evaluated (13). Providers suggested asking about parents’ psychosocial functioning. Assessment of caregiver psychosocial functioning is part of standard care in cystic fibrosis (14) and could be a goal for IBD care. Psychosocial assessment of patients should include areas known to be significantly difficult for youth with IBD (eg, depression, anxiety) (15) as well as social functioning and QOL. Depression and anxiety may be related to disease activity (16) and are 4.6 times higher in adolescent IBD patients (17), with up to 28% having clinically significant depressive or anxiety scores (17–21). According to a meta-analysis, adolescents with IBD were more prone to anxiety, depressive, and internalizing symptoms than other child disease populations (22). Suggested solutions include enhanced population based screening (eg, primary care and subspecialty clinics), cognitive-behavioral therapy (23), peer support groups, and exercise.

Anxiety is common when transitioning (24), and parents expressed anxiety over their child’s transition to a new physician and their child’s self-management, including the disruption in an established medication regimen. Suggested solutions are to begin education at an early age with the development of decision-making, problem-solving, and self-advocacy skills (25), and allowing flexibility in the time of transition (26). A “skills-and-knowledge” checklist approach, as suggested by our parent group, is a solution that some IBD centers have begun implementing. Physicians should begin seeing patients without parents and discuss transition benefits, and the new physician can collaborate with the pediatric physician to transfer of care and educate about the adult health care system (27).

Parent were concerned about limiting college choices to those willing to accommodate their child’s needs. High school teachers were sometimes unsympathetic to bathroom needs or seemed unfamiliar with established 504 plans. While there are limited studies at the high school level, our participants’ perceptions are reinforced by survey research at the college level. Students found colleges unhelpful (6) and made limited or ineffective 504 accommodations (28), and student counselors lacked the necessary training to properly accommodate students (29,30). Through a medical home model, we could provide tools and resources to empower patients and their families to seek assistance when unmet needs or barriers arise. Partnering with the school to provide education and advocacy related to 504 plan implementation is a crucial step.

Providers described struggles to educate families and patients about the seriousness of their disease as part of a strategy to motivate medication adherence. Nonadherence in youth with IBD is significant (31–33). For adolescents, reasons for nonadherence included “lack of time”, medication side effects, “feeling well enough,” and the belief that medications were not working (34). Our study identified adherence barriers including inconvenience/other priorities and social stigma. Suggested strategies to counter intentional nonadherence include educational, behavioral, and cognitive-behavioral interventions, while organizational strategies and reminder systems are suggested for accidental nonadherence (eg, behavioral contracting, reward systems, education, and medication tracking) (35). Patients suggested phone reminders, pill boxes, and developing a plan for taking medications around their activities would help adherence. They also suggested providers offer tips and strategies to help with taking pills, and pharmacies could help by filling multiple prescriptions at the same time to avoid extra trips to the pharmacy.

A common concern among youth in our study was the need to be constantly vigilant when choosing what to eat and this has been shown to negatively affect overall QOL. Prior surveys of adolescents with IBD similarly felt that food restrictions limit their social experiences (36–38) and changed their pleasure in eating (38). Patients suggested healthier food options at school and parents would like clear nutritional recommendations and resources provided by their medical team.

Several parents expressed that providers were unfamiliar with holistic treatments and a lack of emphasis on nutrition as part of treatment, while providers were critical of such families for ignoring their expertise and trusting unreliable sources. Many IBD patients use complementary or alternative medicine to “treat” their IBD, despite the lack of evidence for its effectiveness (39). The provider group recognized a need for ongoing disease education. The parent group suggested a centralized resource hub within the patient portal or a webpage that contains standardized information and checklists and perhaps a newsletter to stay up to date on the disease, treatments, resources, support, and events.

Communication barriers can impact all aspects of care such as scheduling, patient education, adherence, and transferring to adult care. Parents are more likely to accept the risks of therapies if there is a substantial treatment benefit (40). Some of these barriers may be addressed by focusing on disease education and parent education about treatments (41–43), and encouraging patients to ask questions (44). Another solution for improving communication is the use of self-monitoring technology to more faithfully report their real-time symptoms and medication adherence (as long as physicians actively participated by providing feedback) (45). Increasing the use of patient portals and improving timely responses would enhance communication between families and medical teams. Providers suggested a way to enhance communication between primary care and other subspecialists by fostering relationships via education and social networking within the community.

There are several limitations including generalizability, as it reflects the experiences of patients, caregivers, and medical team at a single, large IBD center. This study preferentially recruited patients and families within an hour of the hospital’s main campus location, which could have impacted focus group responses related to geographic location/travel time. This study was conducted prior to the COVID-19 pandemic, which could impact the results of future studies. The focus groups were incredibly helpful identifying barriers; however, identifying actionable solutions for each theme was challenging.

In summary, this qualitative analysis identified several barriers to IBD care, some shared, some unique to patients, parents, and providers, and with varying degrees of tractability which demonstrates the critical importance of obtaining multiple stakeholder perspectives when exploring barriers to care. Some of the high-yield targets across stakeholders include care delay, communication, education, as well as psychosocial and school-related concerns. Some barriers are related to experiences of the disease (eg, bullying and distasteful medications) rather than explicit barriers to medical care, which is a novel aspect of this study. Thus, highlighting the importance of assessing multiple areas of function since they are perceived as barriers to their care. Psychosocial issues should be at the forefront when caring for youth with IBD, and we should include QOL as an essential outcome measure in practice. The QOL/experience aspect of the disease is an important neglected barrier that should be investigated further. In addition, future directions should include a specific, targeted focus on identifying real-world system-level solutions (eg, utilization of the medical home model, tailored resources and processes) to these barriers and expanding the work to include multiple centers and diverse demographics.

## Supplementary Material


