# Supplementary material for: Patient, Parent, and Provider Perceptions of Barriers to Pediatric Inflammatory Bowel Disease Care
Source: JPGN Rep. 2023 Nov 8;4(4):e386. doi: 10.1097/PG9.0000000000000386 (PMC10684132; doi:10.1097/PG9.0000000000000386)
Supplement: Supplementary file 1 [file pg9-4-e386-s001.pdf]

Condensed Parent and Provider Focus Group Discussion Guides

Focus Group Discussion Guide – Parents
"Barriers and Solutions"
Date:

Introduction: Hello, my name is _____________. Thank you for agreeing to participate in this focus group. A lot of people don't receive good health care. This might be related to problems with the healthcare system, such as issues with insurance, prescriptions, clinics, hospitalizations, and communication with doctors. We think patients and their families can help figure out ways to make things better. So we want to learn about any problems you or your family have had with your IBD care.

We will record the groups and keep it confidential. Because we are recording the session, we ask that one person speak at time and to not talk over each other. We also ask that information sharing in the group stays inside the group.

I'm going to be in the background, and I'll give you questions for the group to discuss. There are no right or wrong answers. We want to hear different viewpoints. We are interested in hearing a range of thoughts and experiences, not to develop a consensus. Before we get started, let's take some time to get to know each other.

Icebreaker: [Pair participants] Interview each other and then we will introduce everyone to the rest of the group. What is your name, name and age of your child, and something fun you plan to do this year.
After icebreaker, begin focus group questions/discussion.

Questions:

1. Have you experienced or found it hard to get or understand your care?
2. What challenges do you (or your family) have with getting the medical care he/she needs?

   Probe: You can include items from the past, current, or anticipated future concerns. Some topics might be related to problems with the healthcare system, such as issues with insurance, prescriptions, clinics, hospitalizations, and communication with doctors.

3. What types of solutions (such as resources or changes to the system) would help lessen the barriers/challenges you listed?

   Probe: Mention a couple barriers folks brought up and ask; in what ways can we make those better?

Focus Group Discussion Guide – Providers
"Barriers and Solutions"
Date:

Introduction: Hello, my name is _____________. Thank you for agreeing to participate in this focus group. A lot of people don't receive good health care. This might be related to problems with the healthcare system, such as issues with insurance, prescriptions, clinics, hospitalizations, and communication with the medical team and patients. We think input from medical teams, patients and their families can help figure out ways to make things better. So we want to learn about problems you and/or your patients and their families have had with achieving excellent IBD care.

We will record the groups and keep it confidential. Because we are recording the discussion, it is important that only one person talk at a time. We also ask that information sharing in the group stays inside the group as well.

I'm going to be in the background, and I'll give you questions for the group to discuss. There are no right or wrong answers. We want to hear different viewpoints. Before we get started, let's take some time to get to know each other.

Icebreaker: [Pair participants] Interview each other and then we will introduce everyone to the rest of the group. What is your name, what you do here at Nationwide Children's Hospital, and something that you enjoy about your job?
After icebreaker, begin focus group questions/discussion.

Questions:

1. What barriers or challenges have you experienced with interacting with the health system as part of the medical team?

2. What types of challenges have your patients and their families faced with their IBD care?

Probe: You can include items from the past, current, or anticipated future concerns.

3. What types of solutions (such as resources or changes to the system) would help lessen the barriers/challenges you listed?

Probe: Mention a couple barriers folks brought up and ask; in what ways can we make those better?
